# Predicting thyroid cancer recurrence using supervised CatBoost: A SHAP-based explainable AI approach

**DOI:** 10.1097/MD.0000000000042667

**Published:** 2025-05-30

**Authors:** Ahmad A. Hanani, Turker Berk Donmez, Mustafa Kutlu, Mohammed Mansour

**Affiliations:** a Biomedical and Clinical Basic Skills Department, Faculty of Medicine and Health Sciences, An-Najah National University, Nablus, Palestine; b Biomedical Engineering Department, Sakarya University of Applied Sciences, Sakarya, Turkey.

**Keywords:** CatBoost, explainable AI, glioblastoma multiforme, lower-grade gliomas

## Abstract

Recurrence prediction in well-differentiated thyroid cancer remains a clinical challenge, necessitating more accurate and interpretable predictive models. This study investigates the use of a supervised CatBoost classifier to predict recurrence in well-differentiated thyroid cancer patients, comparing its performance against other ensemble models and employing Shapley Additive Explanations (SHAP) to enhance interpretability. A dataset comprising 383 patients with diverse demographic, clinical, and pathological variables was utilized. Data preprocessing steps included handling values and encoding categorical features. The dataset was split into training and testing sets using a 70:30 ratio. Model performance was evaluated using accuracy and area under the receiver operating characteristic curve. A comparative analysis was conducted with other ensemble methods, such as Extra Trees, LightGBM, and XGBoost. SHAP analysis was employed to determine feature importance and assess model interpretability at both the global and local levels. The supervised CatBoost classifier demonstrated superior performance, achieving an accuracy of 97% and an area under the receiver operating characteristic curve of 0.99, outperforming competing models. SHAP analysis revealed that treatment response (SHAP value: 2.077), risk stratification (SHAP value: 0.859), and lymph node involvement (N) (SHAP value: 0.596) were the most influential predictors of recurrence. Local SHAP analyses provided insight into individual predictions, highlighting that misclassification often resulted from overemphasizing a single factor while overlooking other clinically relevant indicators. The supervised CatBoost classifier demonstrated high predictive performance and enhanced interpretability through SHAP analysis. These findings underscore the importance of incorporating multiple predictive factors to improve recurrence risk assessment. While the model shows promise in personalizing thyroid cancer management, further validation on larger, more diverse datasets is warranted to ensure robustness.

## 1. Introduction

The thyroid gland is a vital endocrine organ located in the anterior part of the neck, and it is responsible for producing hormones that regulate various metabolic processes in the body. It primarily secretes thyroxine (T4) and triiodothyronine (T3), which play critical roles in metabolism, growth, and development. These hormones influence the metabolic rate of cells, affecting how energy is utilized and how tissues develop and function. The thyroid gland’s activity is regulated by the pituitary gland through the secretion of the thyroid-stimulating hormone, ensuring that hormone levels remain balanced to meet the body’s needs.

Thyroid diseases encompass a range of conditions that can disrupt the gland’s normal functioning, with thyroid cancer being a significant concern. Among the various types of thyroid cancer, well-differentiated thyroid cancer (WDTC) is the most common and includes papillary and follicular thyroid carcinomas. These cancers typically have a good prognosis when detected early and treated appropriately, often involving surgical resection followed by radioactive iodine therapy and thyroid hormone suppression therapy. Despite the generally favorable outcomes, understanding the biological behavior and progression of WDTC remains crucial for improving patient management and long-term survival rates.

WDTC encompasses a group of malignancies originating from the thyroid follicular epithelium, predominantly including papillary, follicular, and Hurthle cell carcinomas. These cancers account for the vast majority (95–98%) of all thyroid malignancies and are characterized by their ability to retain certain functional characteristics of normal thyroid cells, such as the production of thyroglobulin and the uptake of iodine. The incidence of thyroid cancer has been on the rise globally, a trend attributed in part to enhanced diagnostic techniques, including high-resolution ultrasound imaging, although this alone does not fully explain the increasing rates observed across various demographics and tumor types.^[1]^

Management strategies for WDTC have been the subject of ongoing debate and evolution, with treatment typically involving a combination of surgical resection, radioactive iodine ablation, and thyroid-stimulating hormone suppression therapy. The extent of thyroidectomy ranging from lobectomy to total thyroidectomy depends on factors such as tumor size, presence of nodal metastasis, and patient risk stratification.^[2]^ Additionally, postoperative radioactive iodine treatment is used to eliminate residual thyroid tissue and to treat microscopic disease, thereby reducing recurrence rates and improving locoregional control.^[3]^

Prognosis for patients with WDTC is generally favorable, with high survival rates, particularly when the disease is detected early and managed appropriately. Risk stratification is crucial in tailoring treatment plans and involves evaluating various clinicopathologic factors to categorize patients into low, intermediate, or high-risk groups. This stratification aids in decision-making regarding the need for additional treatments such as radioactive iodine ablation and the intensity of follow-up regimens.^[4]^

Despite the overall good prognosis, certain subtypes and aggressive forms of WDTC present challenges in management due to variable responses to standard therapies. For instance, Hurthle cell carcinomas and some variants of follicular carcinomas may exhibit less predictable behavior and reduced iodine uptake, complicating treatment efforts.^[5]^ Advances in molecular genetics and the application of machine learning algorithms in risk stratification and prognosis are emerging as valuable tools to enhance the precision of WDTC management.^[1]^

The recurrence of WDTC poses a significant challenge in the clinical setting, affecting a subset of patients even after initial successful treatment. Recurrence can occur locally in the neck or as distant metastases, with various factors influencing the likelihood of recurrence, including the initial tumor stage, lymph node involvement, and patient-specific genetic and molecular characteristics. Monitoring for recurrence involves regular follow-up with clinical examinations, imaging studies, and serum thyroglobulin measurements. Identifying and understanding the variables that contribute to cancer recurrence is essential for developing targeted interventions and improving prognostic models, ultimately enhancing patient outcomes and quality of life.

In parallel with advances in clinical diagnostics and treatment, the use of artificial intelligence (AI) in healthcare has gained substantial momentum. AI methods, particularly those utilizing machine learning algorithms, are being increasingly applied to complex medical problems such as disease prediction, risk stratification, and personalized treatment planning. These approaches have demonstrated potential to complement clinical expertise by offering data-driven insights that are both accurate and scalable. Within the domain of oncology, AI has been particularly valuable in supporting early diagnosis, recurrence prediction, and treatment response evaluation, laying the foundation for more precise and individualized care.

AI has increasingly become integral to healthcare, offering innovative solutions across various medical domains. Wang et al have contributed significantly to this field through multiple studies demonstrating the versatility of AI in clinical diagnostics. In the context of infectious diseases, Wang et al (2024) introduced a stacked deep learning approach for efficient SARS-CoV-2 detection using blood sample analysis, showcasing how AI can enhance diagnostic accuracy during pandemic conditions.^[6]^ In a separate study, Wang et al (2020) proposed a deep learning framework for the early detection of Parkinson disease, focusing on the premotor phase to enable timely intervention.^[7]^ Their model incorporated key clinical indicators such as rapid eye movement disturbances, olfactory dysfunction, cerebrospinal fluid biomarkers, and dopaminergic imaging data. Compared with twelve traditional machine learning and ensemble methods, their deep learning approach achieved superior diagnostic accuracy. Moreover, by leveraging boosting techniques, they provided interpretability through feature importance analysis underscoring the growing emphasis on explainable AI in high-stakes clinical decision-making. Together, these contributions highlight the impactful role of AI in both acute and chronic disease management.

In cardiovascular health, Donmez et al (2024) applied Shapley Additive Explanations (SHAP) and LIME to interpret hypertension risk predictions generated by an XGBoost model trained on clinical and laboratory data.^[8]^ By revealing the influence of key biomarkers on model decisions, the study addressed the limitations of black box AI systems and highlighted the role of explainable machine learning in enhancing transparency. The findings support the use of interpretable AI models for early hypertension detection and more informed risk assessment in clinical practice.

In the field of hematology, Mahmud et al (2023) demonstrated the feasibility of using machine learning for noninvasive anemia detection through lip mucosa image analysis.^[9,10]^ By applying several ML algorithms to color and demographic features, the study showed that anemia can be accurately predicted without the need for blood tests. This approach offers a practical and low-cost alternative for early screening, especially in settings with limited medical resources, highlighting AI’s potential to improve diagnostic accessibility.

Mental health has also benefited from AI advancements. Hanani et al (2024) applied deep and machine learning techniques such as deep neural networks, support vector machines, and random forests (RFs) to predict changes in key psychological indicators including depression, anxiety, and social dysfunction among Palestinian medical students during the COVID-19 pandemic.^[11]^ Their study demonstrated that these models, particularly deep neural networks, could accurately forecast mental health outcomes based on survey data. The findings emphasize the potential of AI to support early identification of mental health risks and enable timely, data-driven interventions.

These studies collectively illustrate the expanding role of AI in healthcare, demonstrating its capacity to enhance diagnostic accuracy, provide interpretable insights, and support noninvasive and predictive health assessments across diverse medical fields. Building on this growing body of evidence, research on thyroid cancer WDTC has increasingly turned toward the use of AI-based models to address challenges in prognosis and recurrence prediction. These methods offer the potential to enhance traditional risk models by integrating diverse clinical, pathological, and demographic variables into cohesive predictive frameworks.

Furthermore, identifying predictive factors for nodal recurrence is crucial for improving patient outcomes in WDTC. A study by Kaur et al (2023) highlights that multifocality, extrathyroidal extension, and high-risk variants are significant predictors of central and lateral compartment nodal recurrence.^[12]^ Their findings underscore the importance of comprehensive surgical and adjuvant treatment plans tailored to these risk factors to mitigate recurrence risks and enhance long-term disease management.

A detailed analysis of thyroid nodule risk assessment was conducted by Pozdeyev et al. Their research demonstrated that combining a deep learning Convolutional Neural Network classifier with a polygenic risk score improved the classification accuracy of thyroid nodules as benign or malignant.^[13]^ Specifically, the area under the receiver operating characteristic curve (AUC) increased from 0.83 to 0.89 (*P* = .007). The combined classifier achieved a sensitivity of 0.95 (95 % confidence interval (CI) [0.88–0.99]), a specificity of 0.63 [0.55–0.70], and positive and negative predictive values of 0.47 [0.41–0.58] and 0.97 [0.92–0.99], respectively. These findings underscore the enhanced diagnostic accuracy gained through the integration of genetic and ultrasound-based assessments.

The study by Lee et al utilized machine learning algorithms to analyze the complications of thyroid damage caused by radiotherapy in patients with head and neck cancer.^[14]^ Their research demonstrated that using the RF algorithm yielded the highest predictive accuracy, with an Area under the AUC of 0.827 and an accuracy of 82.4%. According to their findings, older age and larger thyroid volume were associated with a lower risk of thyroid damage, while higher mean dose, volume of structure (V50), and volume of structure (V60) were linked to an increased risk. These results underscore the significance of these factors in predicting hypothyroidism post-radiotherapy and suggest that the RF algorithm is a valuable tool for clinical decision-making. This study involved 137 patients, and 76.6% of them developed hypothyroidism, with the time range between radiotherapy and hypothyroidism occurrence spanning from 7.2 to 70 months, with a median of 29 months. These insights offer a deeper understanding of the predictors of thyroid dysfunction following radiotherapy in head and neck cancer patients.

In this study, a clinically relevant machine learning model was developed to aid in the prediction of thyroid cancer recurrence, addressing a persistent challenge in endocrine oncology. One of the key medical contributions of this work is its potential to enhance risk stratification, which remains a cornerstone in guiding treatment decisions and follow up intensity for patients with WDTC. Unlike traditional black box algorithms, the integration of explainable AI enables clinicians to visualize and interpret the factors influencing each prediction such as treatment response or lymph node involvement thereby aligning with the growing demand for transparency in clinical decision-support systems. This interpretability is especially critical in oncology, where over or under treatment can significantly affect patient outcomes. By offering a tool that is not only accurate but also interpretable, the study contributes to the evolving field of personalized medicine, supports shared decision-making, and fills a gap in the existing literature where predictive performance often lacks clinical explainability.

## 2. Methodology

This research employs a structured and systematic methodology to investigate the potential of machine learning techniques in predicting differentiated thyroid cancer recurrence. The study utilizes a dataset published by Borzooei and Tarokhian (2023) through the UCI Machine Learning Repository, which provides comprehensive data on demographic, clinical, and pathological variables related to thyroid cancer.^[15]^ The analysis follows a multi-phase process, including data acquisition, preprocessing, model development and training, validation, and interpretation of results.

The primary machine learning algorithm used in this research is the supervised CatBoost classifier, selected for its advanced handling of categorical variables and capability to manage intricate dataset structures effectively. The model’s performance is rigorously evaluated using a suite of metrics, including accuracy, precision, recall, specificity, and F1-score, to ensure a holistic understanding of its effectiveness.

**Figure 1.**
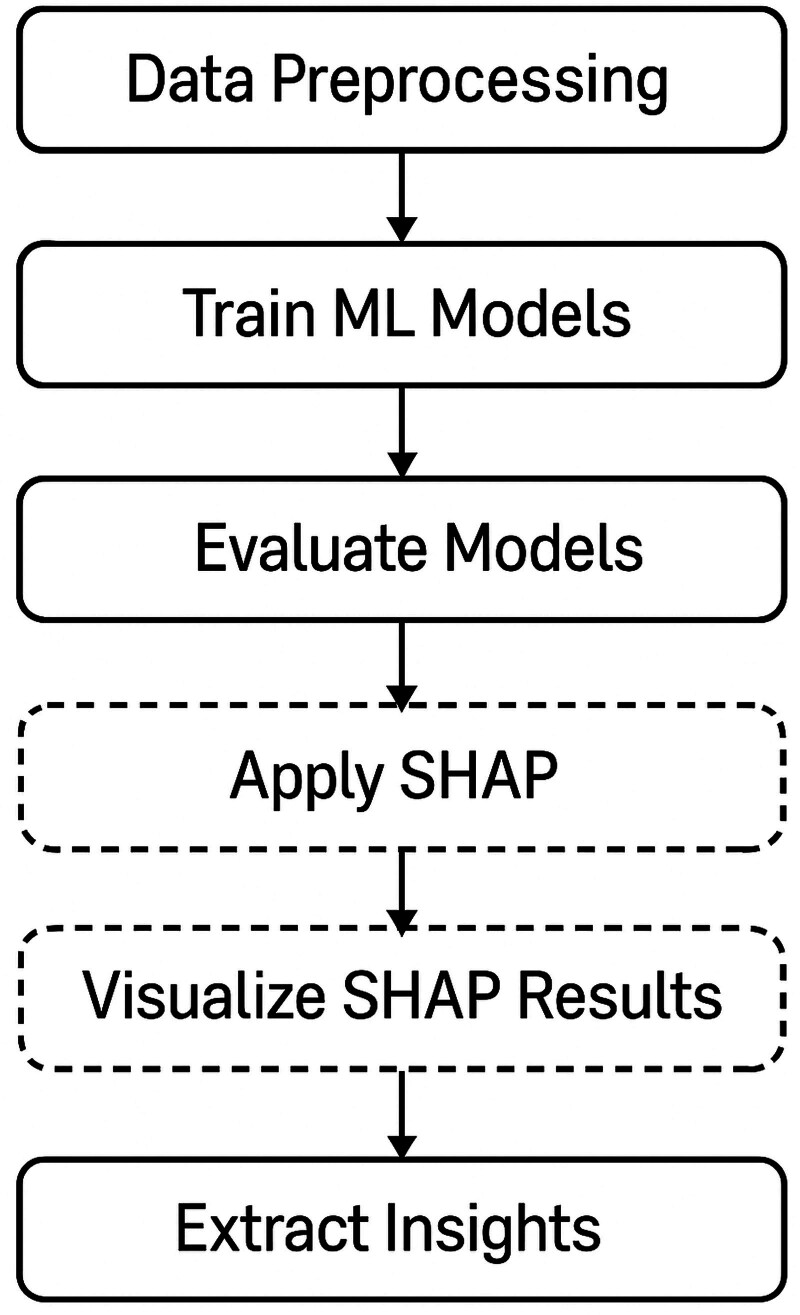
Study flow chart.

In this study, an Explainable Artificial Intelligence (XAI) approach was implemented using SHAP to interpret the predictions of machine learning models. The process began with data preprocessing, which involved data cleaning, feature selection, and splitting the dataset into training and test sets. Multiple machine learning algorithms were trained and evaluated, including Logistic Regression, Random Forest, XGBoost, CatBoost, etc. Based on performance metrics such as accuracy and AUC-ROC, CatBoost was selected as the optimal model for further interpretation due to its superior results (Fig. 1).

To enhance the interpretability of the predictive outcomes, SHAP values are employed. These values provide valuable insights into the importance of various features such as age, gender, history of smoking, thyroid function, pathology, and staging in predicting the recurrence of thyroid cancer. This interpretive layer not only supports the model’s transparency but also highlights the critical factors influencing recurrence.

To explain the predictions of the selected a supervised CatBoost model, SHAP, a model-agnostic and game-theoretic method, was applied. SHAP values were computed using the Tree Explainer module, aligned with feature names, and stored for visualization. Several SHAP-based plots were generated, including summary, dependence, and force/waterfall plots, offering both global and local interpretability. These visualizations enabled the identification of the most influential features, providing deeper insight into model behavior and enhancing transparency. This workflow demonstrates a comprehensive and interpretable machine learning pipeline that supports trust and accountability in data-driven decision-making.

By adopting this methodical approach, the study aims to achieve its objectives with precision, contributing significantly to the understanding of thyroid cancer recurrence. It offers a meaningful advancement in personalized medicine, assisting clinicians in identifying high-risk cases and tailoring treatment strategies to improve patient outcomes.

### 2.1. Data acquisition

This study utilized publicly available datasets that do not contain identifiable patient information. As per institutional guidelines and relevant regulations, ethical approval was not required. The foundational dataset for this study was selected from the “Differentiated Thyroid Cancer Recurrence” dataset curated by Borzooei and Tarokhian and hosted by the UCI Machine Learning Repository. This dataset serves as a vital resource for exploring the clinical and pathological factors influencing thyroid cancer recurrence. It comprises 383 cases, offering a robust basis for predictive modeling and recurrence analysis undertaken in this research, with no missing values present in the dataset (Table 1).

**Table 1:** Gender and recurrence information in the thyroid dataset.

Gender	No recurrence	Recurrence	Total
Female	246	66	312
Male	29	42	71
Total	275	108	383

A comprehensive breakdown of the dataset reveals the gender distribution and recurrence rates, providing a balanced and representative demographic of individuals affected by differentiated thyroid cancer. This diversity supports the development and validation of models with increased generalizability.

The dataset selection criteria prioritized the inclusion of detailed clinical and demographic data, such as gender and recurrence status, to ensure robust and accurate analyses. By leveraging this well-structured dataset, the study aims to improve diagnostic precision and develop personalized treatment approaches in the management of thyroid cancer recurrence.

### 2.2. Data preprocessing

Upon securing the differentiated thyroid cancer dataset from the UCI Machine Learning Repository as curated by Borzooei and Tarokhian, an extensive data preprocessing phase was undertaken to ensure the dataset’s readiness for subsequent analysis. This phase was critical in maintaining data quality and uniformity, thereby underpinning the model’s predictive accuracy.

The preprocessing journey began with the identification and handling of values, a crucial step given the clinical nature of the dataset. To preserve the integrity of the analysis, statistical imputation techniques were employed where necessary, while records essential clinical information were excluded to maintain the robustness of the study.

The dataset prominently features categorical variables, including demographic information such as gender (encoded as 0 for Female and 1 for Male) and recurrence status (encoded as 0 for No Recurrence and 1 for Recurrence). These variables were carefully encoded into a numerical format suitable for machine learning models, ensuring their clinical relevance was retained (Table 2).

**Table 2:** Legend of the differentiated thyroid cancer dataset.

Description	Variable name	Value type
Gender	Gender	0: Female, 1: Male
Age	Age	Continuous
Smoking status	Smoking	0: nonsmoker, 1: Smoker
Smoking history	Hx	0: No history, 1: History
History of radiotherapy	Hx Radiotherapy	Radiotherapy 0: No, 1: Yes
Thyroid function	Thyroid Function Thyroid Function 0: Normal, 1: Abnormal	Thyroid Function Thyroid Function 0: Normal, 1: Abnormal
Physical examination findings	Physical Examination	0: No findings, 1: Findings present
Adenopathy	Adenopathy	0: Absent, 1: Present
Pathology results	Pathology Results	0: Negative, 1: Positive
Focality	Focality	0: Unifocal, 1: Multifocal
Risk level	Risk	0: Low, 1: Intermediate, 2: High
Tumor (T)	T	0: T0, 1: T1, 2: T2, 3: T3, 4: T4
Node (N)	N	0: N0, 1: N1, 2: N2, 3: N3
Metastasis (M)	M	0: M0, 1: M1
Stage	Stage	0: Stage 0, 1: Stage I, 2: Stage II, 3: Stage III, 4: Stage IV
Treatment response	Response	0: No response, 1: Partial, 2: Stable disease, 3: Complete response
Recurrence	Recurred	0: No recurrence, 1: Recurrence

Normalization was applied to continuous variables like age to mitigate scale and variance discrepancies, ensuring no feature disproportionately influenced the model due to its magnitude. This step enhanced the dataset’s balance and consistency across all variables.

Additionally, binary indicators for clinical and pathological features such as smoking history, history of radiotherapy, thyroid function, and other factors were included, providing a comprehensive dataset for analysis.

Finally, the dataset was partitioned into training and testing sets using a 70:30 ratio. This division was executed to ensure that both subsets accurately reflected the diversity within the dataset, encompassing demographic variations, clinical features, and recurrence status, thereby facilitating the model’s evaluation on previously unseen data.

These meticulous preprocessing steps transformed the dataset into an optimal format for high-quality machine learning analysis, laying a strong foundation for the development and evaluation of the predictive model.

### 2.3. Model development and training

Following the meticulous preprocessing of the differentiated thyroid cancer dataset, the focus shifted to model development and training, with an emphasis on balancing predictive accuracy and interpretability. Among the various algorithms evaluated, a supervised CatBoost stood out for its superior handling of categorical variables and its compatibility with SHAP for interpreting predictions. Although not the most accurate model, supervised CatBoost was prioritized due to its ability to provide insights into the decision-making process (Table 3).

**Table 3:** Model performance metrics.

Model	Accuracy	Precision	Recall	F1 score	AUC	Log loss
Explainable Boosting Machine	0.97 0.97	0.97	0.97	0.97	0.99	0.98
A supervised CatBoost	0.97	0.97	0.97	0.97	0.99	0.98
Extra Trees	0.96	0.96	0.96	0.96	0.99	0.97
LightGBM	0.96	0.96	0.96	0.96	0.99	0.99
Adaboost	0.96	0.96	0.96	0.96	0.99	0.97
XGBoost	0.96	0.96	0.96	0.96	0.99	0.99
Random Forest	0.96	0.96	0.96	0.96	0.99	0.98
Decision Tree	0.93	0.93	0.94	0.93	0.94	0.80
Logistic Regression	0.90	0.90	0.89	0.90	0.93	0.90
Naive Bayes	0.88	0.87	0.88	0.88	0.96	0.93
KNN	0.88	0.87	0.87	0.88	0.91	0.80
Support Vector Machine	0.79	0.74	0.84	0.79	0.91	0.80

The supervised CatBoost classifier was initialized with hyperparameters designed to optimize its performance for predicting thyroid cancer recurrence. Despite its accuracy not being the highest among all models, careful tuning and the use of the evaluation metric “Accuracy” ensured a balance between computational efficiency and predictive reliability.

The training phase utilized 70% of the preprocessed dataset, allowing CatBoost to learn the relationships between clinical and demographic features and recurrence status. This phase emphasized computational efficiency while minimizing prediction error, with verbose outputs suppressed to streamline the process.

SHAP values were integrated post-training to explain the contributions of individual features to the model’s predictions. This step was critical not only for validating the utility of CatBoost but also for ensuring the model’s outputs were interpretable and clinically actionable. By leveraging SHAP, the study reinforced its commitment to transparency and the practical application of machine learning in thyroid cancer management.

Upon completion of the training, a supervised CatBoost demonstrated impressive performance, achieving metrics comparable to the most accurate models while offering superior interpretability. The table below highlights the comparative performance of the evaluated models.

This balanced approach, emphasizing both performance and interpretability, provides a solid foundation for the validation phase. It ensures the model’s predictions are not only accurate but also understandable, aligning with the study’s goals of clinical relevance and actionability.

### 2.4. Model validation

A portion of the dataset was reserved for validation to assess the model’s predictive performance critically. This validation phase employed a stratified sampling strategy to ensure a balanced representation of recurrence and non-recurrence cases across both genders. Key performance metrics, including accuracy, precision, recall, F1-score, and AUC, were calculated to evaluate the model’s effectiveness.

The supervised CatBoost classifier, a gradient boosting algorithm specifically designed for categorical data, was highlighted for its efficient handling of these features. Unlike traditional methods, it eliminates the need for preprocessing steps like one-hot encoding or label encoding, making it particularly suited for datasets with diverse categorical variables.^[16]^

The gradient boosting mechanism in a supervised CatBoost optimizes an objective function that combines a loss function and a regularization term:


$$\begin{array}{l} L\left(y,F\right)=\underset{i=1}{\overset{N}{\sum }}\;l\left(y_{i},F\left(x_{i}\right)\right)+\underset{k=1}{\overset{K}{\sum }}\;\Omega \left(f_{k}\right), \end{array}$$

where y represents the true labels, F is the ensemble model, l is a differentiable convex loss function, $f_{k}$ are the individual trees, and Ω is the regularization term. The algorithm’s inherent support for categorical data and its integration with SHAP values for interpretability are key advantages.^[17]^

In addition to its explainability, the CatBoost model demonstrated strong performance during training and cross-validation. The 5-fold cross-validation yielded consistently high accuracy scores across all folds, with 97.40%, 94.81%, 96.10%, 98.68%, and 94.74%, respectively. The mean accuracy was calculated as 96.35%, with a standard deviation of 1.52%, highlighting the model’s stability and reliability in predicting outcomes.

**Figure 2.**
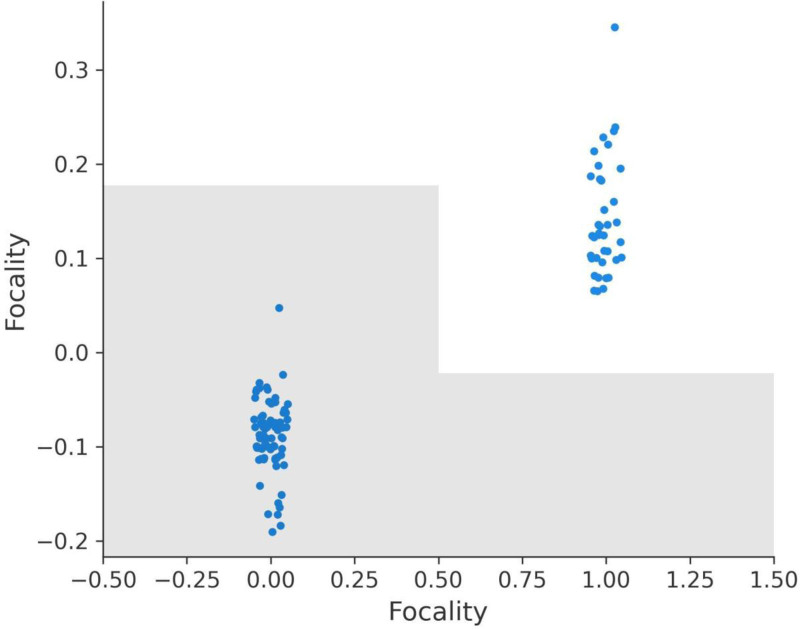
Confusion matrix.

**Figure 3.**
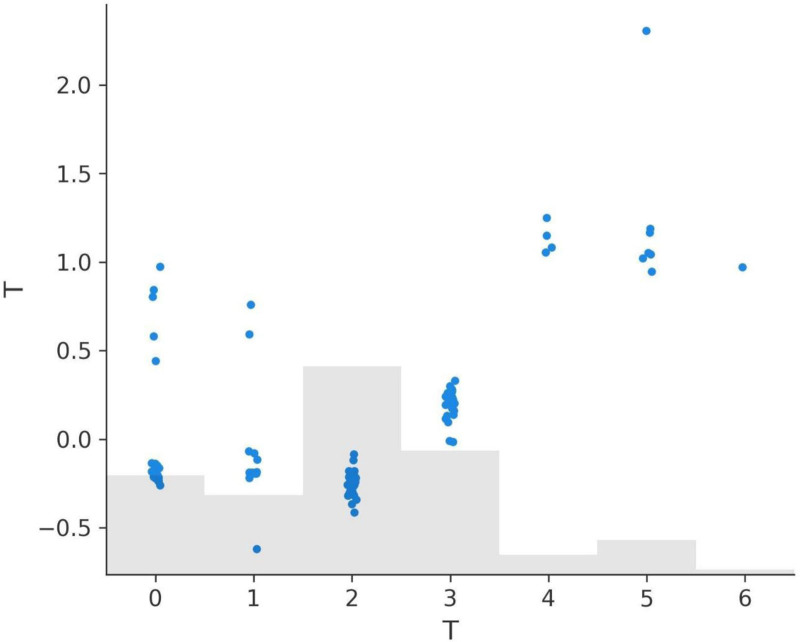
ROC curve.

During validation, the supervised CatBoost model demonstrated strong classification capabilities, achieving an area under the ROC value of 0.97, showcasing its excellent discriminative power in distinguishing recurrence cases. The confusion matrix further supports these results, with the following values: true negatives (TN): 81, false positives (FP): 2, false negatives (FN): 2, and true positives (TP): 30. These metrics underscore the model’s precision and reliability in predicting recurrence (Figs. 2 and 3).

### 2.5. Model interpretation with SHAP

SHAP is a powerful framework designed to provide insights into the predictions of any machine learning model, enabling a deeper understanding of how features contribute to decision-making. Introduced in 2017, SHAP is grounded in cooperative game theory and aims to unify diverse approaches to model interpretability under a single, consistent methodology. By assigning an “importance value” to each feature, SHAP quantifies the impact of individual features on specific predictions, offering a transparent and accurate explanation of model behavior.^[18]^

At the core of SHAP lies the concept of the Shapley value, a principle derived from cooperative game theory. Shapley values were originally developed to distribute gains or costs among participants in a cooperative setting. In the context of machine learning, SHAP leverages this concept to fairly attribute the contribution of each feature to a model’s prediction. For a given feature, the SHAP value is calculated by considering all possible subsets of features that exclude the feature in question and comparing the model’s predictions with and without the feature included. The mathematical formulation for the SHAP value of a feature $f_{i}$ is expressed as:


$$\begin{array}{l} f_{i}=\underset{S\subseteq F\backslash \left\{i\right\}}{\sum }\;\frac{|S|!\cdot \left(|F|-|S|-1\right)!}{|F|!}\left[f_{S\cup \left\{i\right\}}(x_{S\cup \left\{i\right\}})-f_{S}(x_{S})\right] \end{array}$$

In this equation, F represents the set of all features, S denotes a subset of F that excludes the i th feature, $f_{S\cup \left\{i\right\}}$ and $f_{S}$ correspond to the model’s predictions using the feature sets S∪{i} and S, respectively, while $x_{S\cup \left\{i\right\}}$ and $x_{S}$ represent the associated feature values.

Although the exact computation of SHAP values is theoretically sound, it is computationally expensive, especially for datasets with a large number of features. This computational complexity arises because the calculation involves evaluating the contribution of a feature across all possible subsets of features, a process that grows exponentially with the number of features. To overcome this limitation, SHAP incorporates efficient approximation techniques such as Shapley sampling and Shapley quantitative influence. These methods reduce the computational burden while maintaining the interpretative power of the SHAP framework.

SHAP values can be interpreted from both a global and local perspective, offering versatility in analyzing model behavior. From a global viewpoint, SHAP provides an overview of feature importance across the entire dataset. Features with high absolute SHAP values across multiple samples are identified as having significant influence on the model’s predictions. This global analysis is instrumental in understanding the overall trends and key drivers in the dataset. Conversely, from a local perspective, SHAP values offer granular insights into individual predictions. By explaining how specific features contributed to a particular prediction, SHAP facilitates a transparent understanding of model decisions at the sample level. This dual perspective ensures that SHAP is not only useful for model interpretability but also for gaining actionable insights in real-world applications.^[19]^

The SHAP framework’s ability to unify interpretability methods, its solid foundation in game theory, and its provision for both global and local analysis make it an indispensable tool in modern machine learning workflows. By bridging the gap between complex model outputs and human understanding, SHAP empowers data scientists and domain experts to build more reliable and transparent predictive models.

## 3. Results

The analysis within this section delves into the efficacy of the supervised CatBoost algorithm applied to the glioma dataset, employing SHAP values to elucidate the model’s interpretive capabilities and the influence of distinct features on its predictions.

### 3.1. Global SHAP values analysis

The TNM staging system is a globally recognized framework used to describe the extent and severity of cancer.^[20]^ It comprises 3 components: T (Tumor), N (Node), and M (Metastasis). The T category defines the size and extent of the primary tumor, ranging from T0 (no tumor evidence) to T4 (large tumor with significant invasion). The N category assesses lymph node involvement, with N0 indicating no lymph node metastasis and N3 representing extensive lymph node spread. Lastly, the M category describes the presence of distant metastases, with M0 indicating no metastases and M1 denoting distant metastatic disease. The TNM staging system provides a standardized way to evaluate cancer progression, aiding clinicians in prognosis, treatment planning, and patient stratification.

The global SHAP analysis highlights the most influential features impacting the model’s predictions for thyroid cancer recurrence.^[18]^ Response, representing the treatment outcome, is the most significant predictor, with a SHAP value of 2.077. This underscores its critical role in determining recurrence likelihood. Risk, a categorization of patients based on recurrence probability, follows with a SHAP value of 0.859, emphasizing its importance in risk assessment. N (lymph node involvement), with a SHAP value of 0.596, further highlights the impact of lymphatic spread on recurrence predictions.

T (tumor size) and age are also key contributors, with SHAP values of 0.342 and 0.304, respectively. These factors reflect the model’s ability to incorporate tumor dimensions and patient demographics into its predictions. Adenopathy, which identifies swollen lymph nodes, has a SHAP value of 0.271, while gender, representing biological sex, contributes with a value of 0.219. Stage, a comprehensive measure of disease progression, has a SHAP value of 0.149.

Other significant features include Physical Examination (0.121), indicating the role of clinical findings, and Focality (0.103), which differentiates between unifocal and multifocal tumors. Thyroid Function (0.050), Pathology (0.048), and smoking-related variables (Smoking at 0.012 and Hx Smoking at 0.008) provide additional context, though their contributions are less pronounced. Features such as M (distant metastases, 0.005) and Hx Radiotherapy (history of radiation treatment, 0.001) exhibit minimal influence.

**Figure 4.**
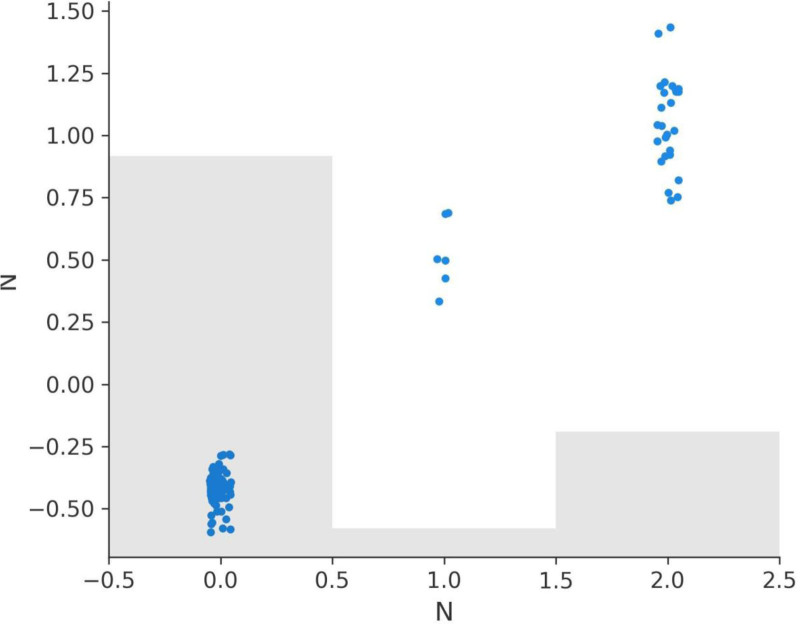
Global SHAP values showing the relative importance of features in predicting thyroid cancer recurrence.

The global SHAP values reveal the hierarchical importance of features in predicting recurrence. Response, Risk, and N emerge as the dominant predictors, while other variables contribute nuanced insights into the recurrence process. This analysis enhances the interpretability of the model, offering clinicians actionable insights for patient evaluation and treatment planning. The hierarchical importance of these features is illustrated in Figure 4.

### 3.2. Local SHAP values analysis

The granular analysis of local SHAP values for individual patient instances provides critical insights into the supervised CatBoost model’s interpretability and performance in predicting thyroid cancer recurrence. By examining specific cases categorized as TP, TN, and FP, we can better understand the contributions of key features to the model’s predictions. This level of analysis validates the model’s efficacy in correctly classifying cases while highlighting areas for refinement by identifying reasons behind any inaccuracies.

Table 4 presents a transposed view of feature values for selected patient instances. This format emphasizes the differences and similarities in clinical and demographic details across patients in different predictive categories (Table 4).

**Table 4:** Actual values from dataset for selected patient instances.

Feature	Patient 324 (TP)	Patient 210 (FP)	Patient 285 (TN)	Patient 225 (TP)
Age	79	28	62	62
Gender	1	0	0	0
Smoking	1	0	0	0
Hx smoking	0	0	0	0
Hx radiotherapy	0	0	0	0
Thyroid function	0	0	0	2
Physical examination	2	2	1	2
Adenopathy	2	1	0	0
Pathology	0	0	3	0
Focality	1	0	1	0
Risk	1	1	1	0
T	3	2	3	2
N	2	2	0	0
M	0	0	0	0
Stage	1	0	1	0

This transposed table provides a side-by-side comparison of selected instances, making it easier to identify patterns and differences in feature values across different predictive categories. By integrating this information with local SHAP analyses, we gain a clearer understanding of how individual features influence the model’s decisions. The analysis demonstrates the model’s capacity to correctly classify instances, as seen in TP and TN cases, while also revealing areas for improvement in false predictions like FP. This approach reinforces the value of explainable AI in clinical applications, enhancing transparency and trust in predictive models.^[8]^

### 3.3. TP case analysis: Instance 324

The examination of a TP instance, specifically patient index 324, provides a detailed understanding of the supervised CatBoost model’s decision-making process when correctly predicting recurrence. The local SHAP values reveal the contributions of specific features to the model’s prediction, offering a granular perspective on the factors driving its classification.

Interestingly, the Response feature, while indicative of positive outcomes, contributes minimally to the recurrence prediction for this instance. This suggests that although the patient had a favorable treatment response, the model predicted recurrence correctly because other features presented substantial evidence indicative of recurrence risk. This highlights the model’s ability to integrate multiple features and balance their contributions effectively.

The Age feature emerges as the most significant contributor, with a SHAP value of 0.909, strongly influencing the model’s prediction towards recurrence. This underscores the critical role of patient age in identifying recurrence risk, highlighting its importance as a key demographic predictor.

Risk level follows closely with a SHAP value of 0.851, reflecting the model’s reliance on stratified patient risk categories to inform its predictions. Similarly, the N (lymph node involvement) feature has a SHAP value of 0.820, emphasizing the importance of lymphatic spread in recurrence prediction.

Additional influential features include Stage (0.640), Adenopathy (0.388), and Gender (0.280). These clinical and demographic factors collectively shape the model’s understanding of recurrence likelihood. While T (tumor size, 0.116) and Focality (0.101) also contribute meaningfully, their impact is relatively smaller compared to the top predictors.

Interestingly, features like Smoking (0.068) and Thyroid Function (0.054) show lower SHAP values but still play a role in refining the model’s decision. These minor contributions illustrate the nuanced interplay of clinical and lifestyle factors in predicting recurrence.

**Figure 5.**
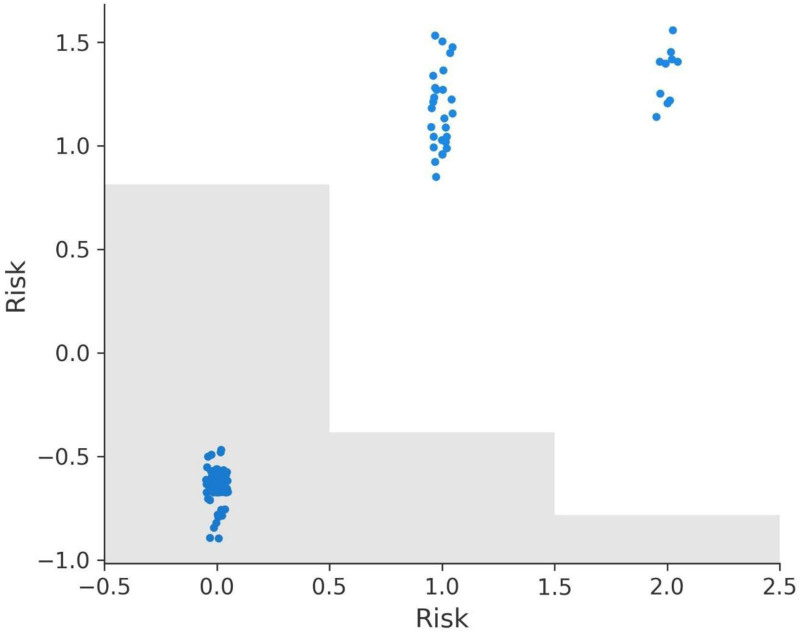
Local SHAP values for patient 324 (true positive), highlighting the contributions of key features to the prediction.

The SHAP plot for instance 324, presented in Figure 5, visualizes these feature contributions, clearly illustrating their relative impact on the model’s prediction. The correct classification of this instance as a recurrence case affirms the model’s capacity to integrate diverse features effectively, providing a robust and interpretable framework for clinical decision-making (Fig. 5).

This case analysis demonstrates the model’s ability to utilize a combination of demographic, clinical, and pathological factors to make accurate predictions. The inclusion of Response as a counterbalancing feature highlights the complexity of the decision-making process, where the model successfully identifies recurrence despite favorable individual factors. Understanding these contributions provides valuable insights for clinicians, paving the way for more targeted and personalized approaches in thyroid cancer management.

### 3.4. TN case analysis: Instance 210

The examination of a TN instance, specifically patient index 210, provides a detailed understanding of the supervised CatBoost model’s decision-making process when correctly predicting the absence of recurrence. The local SHAP values reveal the contributions of individual features to the model’s prediction, offering insights into the factors that led to the correct classification.

The Response feature, with a significant SHAP value of -2.543, emerges as a dominant factor driving the prediction towards non-recurrence. This substantial negative SHAP value indicates that the patient’s favorable treatment response strongly influenced the model’s confidence in predicting the absence of recurrence. The inclusion of this feature highlights the model’s ability to effectively incorporate treatment outcomes into its predictions.

The N (lymph node involvement) feature is another major contributor, with a SHAP value of 1.199, pushing the model’s prediction towards the non-recurrence category. This underscores the critical role of lymph node status in the model’s decision-making process, aligning with its established importance in recurrence risk assessment.

The Risk level follows closely with a SHAP value of 1.182, indicating its significant influence in affirming the prediction of non-recurrence. This feature highlights the model’s reliance on stratified risk categories to guide its predictions effectively. Adenopathy (presence of swollen lymph nodes) also plays a meaningful role, contributing a SHAP value of 0.482 towards the prediction.

Other features, such as Focality (0.048), have smaller positive SHAP values, suggesting they contribute to refining the prediction but are less impactful compared to the top 3 features. Conversely, features such as Hx Radiotherapy (-0.001), M (-0.004), and Smoking (-0.013) exert negative SHAP values, indicating they push the prediction slightly towards recurrence but are overshadowed by the dominant positive contributions of Response, N, Risk, and Adenopathy.

**Figure 6.**
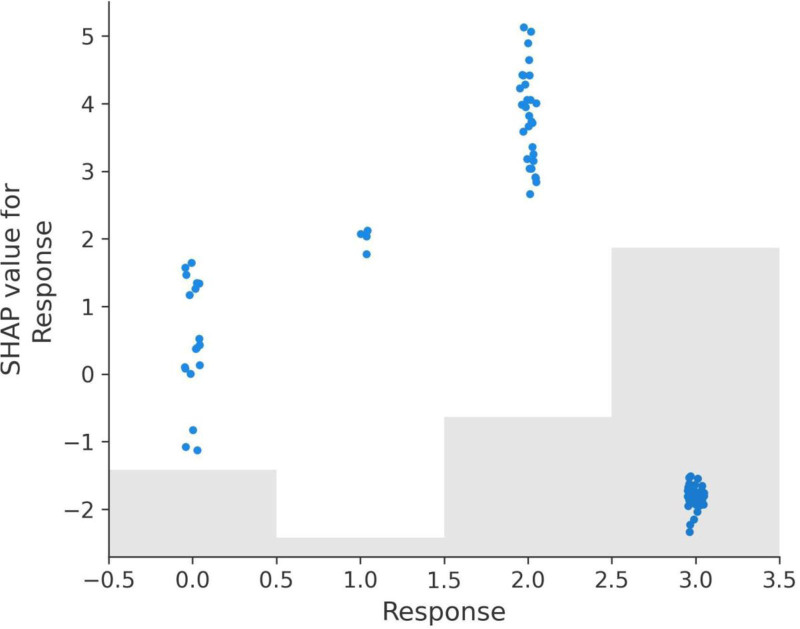
Local SHAP values for patient 210 (true negative), highlighting the contributions of key features to the prediction.

The SHAP plot for instance 210, presented in Figure 6, visualizes these feature contributions, clearly illustrating their relative impact on the model’s prediction. This visualization underscores the model’s capability to leverage strong positive signals from critical features while mitigating the minor conflicting effects of less influential features (Fig. 6).

This case analysis demonstrates the model’s robustness in identifying the absence of recurrence by relying on key clinical and pathological features. The strong contribution of the Response feature, combined with other critical variables like N, Risk, and Adenopathy, highlights the model’s capacity to integrate diverse factors into its predictions. By examining local SHAP values, clinicians can gain deeper insights into the factors influencing individual predictions, fostering transparency and trust in the model’s application to real-world scenarios.

### 3.5. False positive (FP) case analysis: Instance 285

The examination of a FP instance, specifically patient index 285, offers critical insights into the supervised CatBoost model’s misclassification processes. In this case, the model incorrectly predicted recurrence for a patient who did not experience recurrence. The local SHAP values reveal the contributions of individual features to the prediction, highlighting the factors that led to this erroneous classification.

The Risk feature emerges as the most significant contributor, with a SHAP value of 1.363, heavily influencing the model’s prediction toward recurrence. This high SHAP value suggests that the model overrelied on the patient’s risk category, potentially misjudging its impact in this specific instance.

Age is the second most influential feature, with a SHAP value of 0.942, further pushing the prediction toward recurrence. This demonstrates the model’s sensitivity to patient age, which, while generally important, may have been overestimated in this case. The Stage feature also plays a significant role, contributing a SHAP value of 0.896, further reinforcing the model’s confidence in predicting recurrence.

Additional features such as Physical Examination (0.201) and Response (0.130) provided moderate contributions to the recurrence prediction. Interestingly, Response, typically a counterbalancing feature favoring non-recurrence, contributed positively in this instance. This anomaly suggests that the model may have misinterpreted the relationship between treatment response and recurrence likelihood in this specific case.

On the other hand, features like Hx Radiotherapy (-0.000) and M (-0.002) provided negligible negative contributions, indicating minimal influence in countering the strong positive signals from Risk, Age, and Stage.

**Figure 7.**
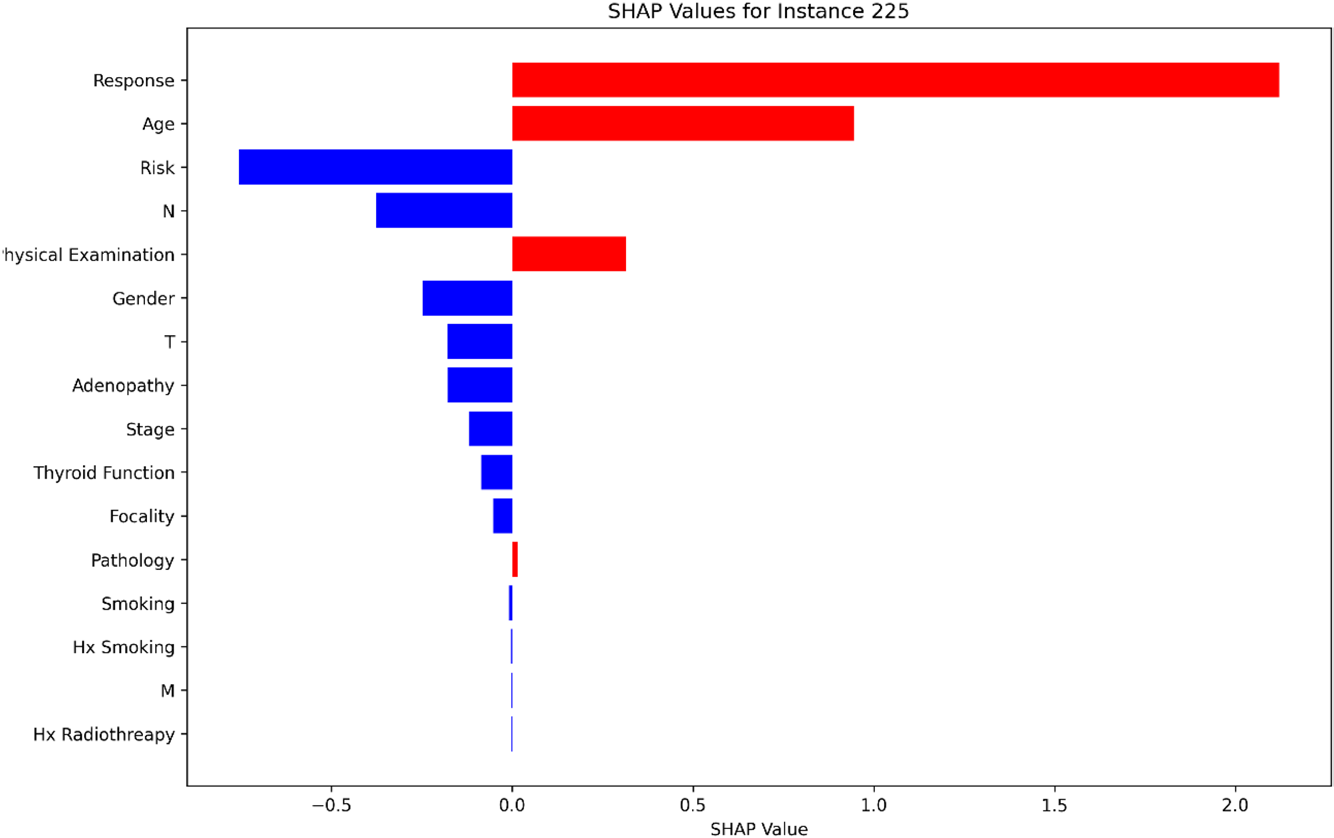
Local SHAP values for patient 285 (false positive), highlighting the contributions of key features to the incorrect prediction.

The SHAP plot for instance 285, presented in Figure 7, visualizes these feature contributions, clearly illustrating their relative impact on the model’s prediction. This visualization underscores the overemphasis on certain features and the underutilization of counterbalancing factors, leading to the misclassification (Fig. 7).

This case analysis highlights the challenges faced by the model in balancing strong signals from key features like Risk, Age, and Stage with other contextual factors. The misclassification underscores the need for further refinement in the model’s training process to better account for complex interactions and reduce the likelihood of FPs. Understanding such cases provides valuable insights into areas where the model can be improved, ultimately enhancing its reliability and applicability in clinical decision-making.

### 3.6. False negative (TN) case analysis: Instance 225

The examination of a FN instance, specifically patient index 225, offers critical insights into the supervised CatBoost model’s failure to predict recurrence for a patient who actually experienced it. The local SHAP values reveal the contributions of individual features to the prediction, highlighting the factors that led to this misclassification.

The Response feature emerges as the most significant contributor, with a SHAP value of 2.121, pushing the prediction strongly toward non-recurrence. This indicates that the patient’s favorable treatment response heavily influenced the model’s confidence, ultimately overriding other features indicative of recurrence. This overreliance on Response underscores a potential area for refinement in the model’s training.

Risk, with a SHAP value of -0.814, strongly supported a recurrence prediction. As a critical variable in assessing the likelihood of recurrence, its significant negative SHAP value indicates that the model correctly identified this as an important factor. However, its influence was insufficient to counteract the dominant positive contribution of Response.

Similarly, the N (lymph node involvement) feature, with a SHAP value of -0.517, also contributed toward recurrence prediction. While this is an important factor in recurrence risk, its relatively weaker influence compared to Response limited its ability to shift the model’s prediction.

Age, with a SHAP value of 0.945, slightly pushed the prediction toward recurrence but could not compensate for the strong positive contribution from Response. The Physical Examination feature, contributing a SHAP value of 0.314, further supported the recurrence prediction.

However, other features, such as Pathology (0.015), provided negligible positive contributions, while features like Thyroid Function (-0.086), Focality (-0.052), Smoking (-0.009), and Hx Smoking (-0.003) exerted minor negative contributions, collectively tilting the prediction further toward non-recurrence.

**Figure 8.**
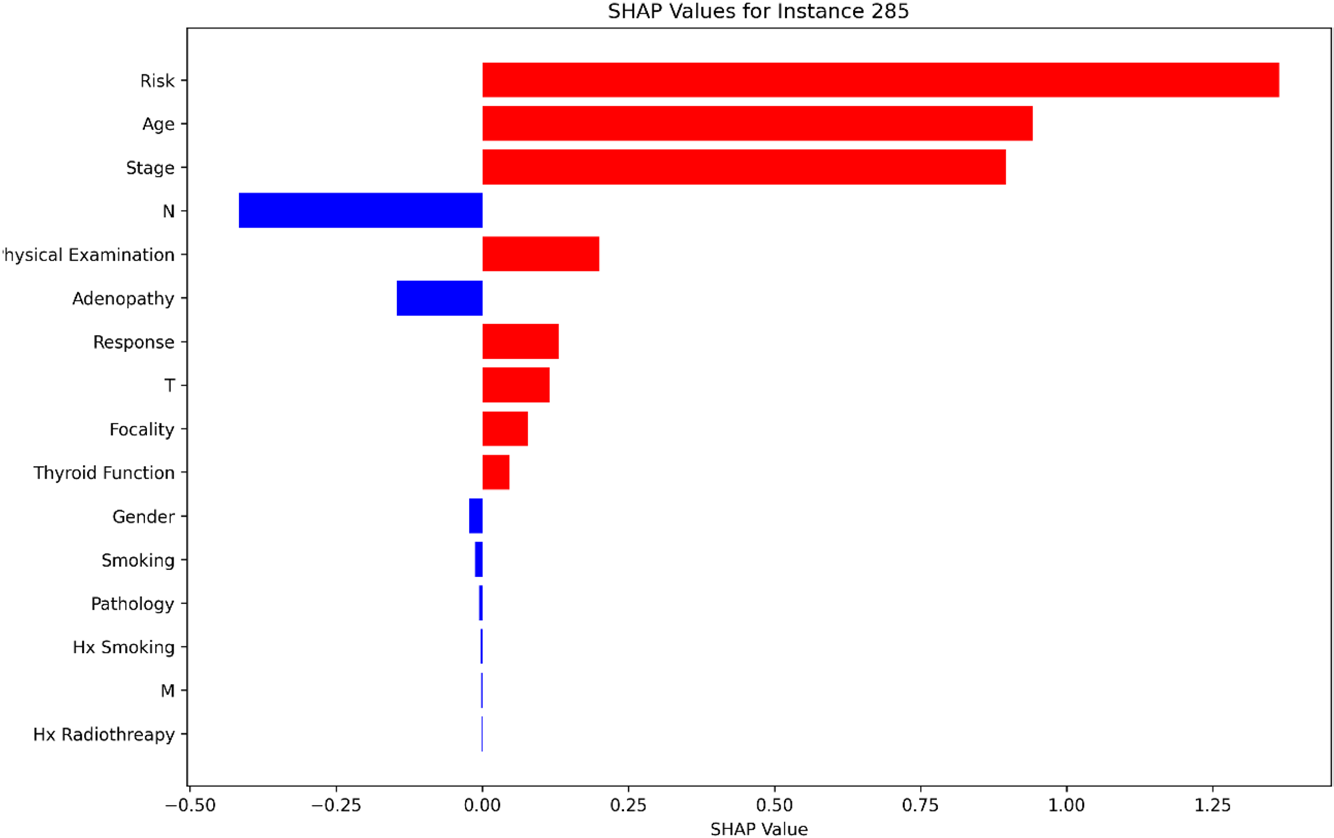
Local SHAP values for patient 225 (false negative), highlighting the contributions of key features to the incorrect prediction.

The SHAP plot for instance 225, presented in Figure 8, visualizes these feature contributions, illustrating how the dominant positive SHAP value for Response overshadowed the significant contributions from Risk and N, ultimately leading to the misclassification (Fig. 8).

This case analysis highlights the challenges faced by the model in correctly identifying recurrence when a single feature, such as Response, exerts a disproportionately strong influence. The misclassification underscores the importance of refining the model to better account for the interactions between features and to mitigate overreliance on dominant variables like Response. Understanding such cases provides valuable insights into areas where the model can be improved, ultimately enhancing its reliability and clinical applicability.

### 3.7. SHAP dependence plots for variables

The SHAP dependence plots for various features offer critical insights into how these features influence the model’s predictions. These plots illustrate the relationship between each feature’s values and their corresponding SHAP values, providing an interpretable framework for understanding the model’s behavior.

**Figure 9.**
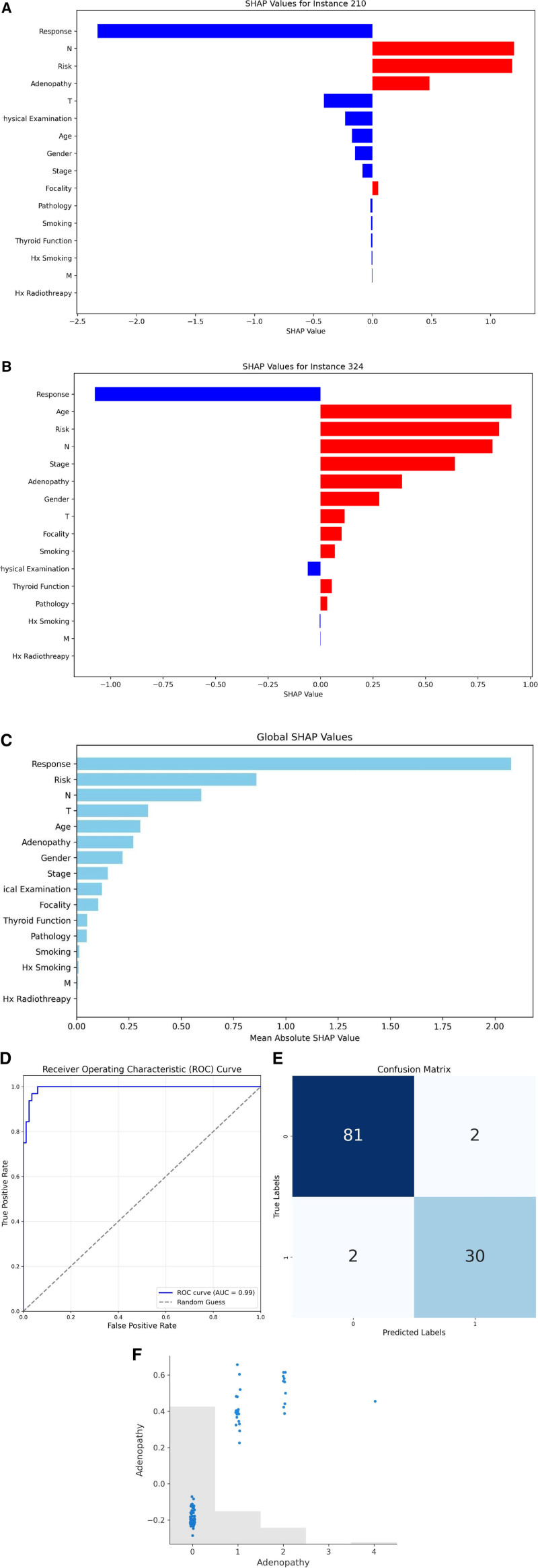
SHAP dependence plots for key features. (A) Response dependence plot, (B) N dependence plot, (C) risk dependence plot, (D) T dependence plot, (E) focality dependence plot, (F) adenopathy dependence plot.

The Response feature demonstrates the most substantial impact on the model’s predictions, with SHAP values ranging from -2.330 to 5.135 (Fig. 9a). A response value of 3, indicating complete recovery, does not contribute to recurrence predictions as it aligns with negative SHAP values. However, lower response values of 0 (no response), 1 (partial response), and 2 (stable disease) progressively contribute to recurrence, with SHAP values indicating that 2 has a stronger effect than 1, and 1 has a stronger effect than 0.

The N (lymph node involvement) feature also shows a significant impact, with SHAP values ranging from -0.593 to 1.436 (Fig. 9b). N0, indicating no lymph node involvement, does not contribute to recurrence predictions, aligning with negative SHAP values. However, values of N1, N2, and N3 progressively increase recurrence predictions, with higher stages having a greater positive impact on SHAP values and recurrence likelihood.

The Risk feature exhibits a wide range of SHAP values, from -0.894 to 1.558 (Fig. 9C). Patients classified as low risk (value 0) have SHAP values aligned with non-recurrence predictions. However, medium risk (value 1) and high-risk (value 2) progressively contribute to recurrence, with high risk having the most pronounced positive SHAP values, emphasizing its role in recurrence stratification.

The T (tumor size) feature, with SHAP values ranging from -0.617 to 2.306 (Fig. 9D), shows that T0, T1, and T2 stages have little to no positive effect on recurrence predictions. However, higher stages such as T4, T5, and T6 are associated with positive SHAP values, indicating their contribution to recurrence likelihood and reflecting the clinical understanding that larger tumor sizes are more likely to lead to recurrence.

The Focality feature shows SHAP values ranging from -0.190 to 0.346 (Fig. 9E). Unifocal disease (value 0) does not contribute to recurrence predictions and aligns with negative SHAP values. Multifocal disease (value 1), on the other hand, contributes positively to recurrence predictions, reinforcing its association with higher recurrence risk.

Lastly, the Adenopathy feature has SHAP values ranging from -0.217 to 0.482 (Fig. 9F). The absence of adenopathy (value 0) does not contribute to recurrence predictions. However, the presence of adenopathy (value 1) contributes positively to recurrence predictions, with its effect varying based on the severity of the condition.

In summary, the SHAP dependence plots provide valuable insights into the model’s behavior and the importance of key features in recurrence prediction. Response, Risk, N, and T have the most pronounced effects, aligning closely with clinical expectations. Focality and Adenopathy also play significant roles, highlighting multifocal disease and adenopathy presence as factors increasing recurrence likelihood. These insights can guide clinical decision-making and treatment strategies, underscoring the interpretability and utility of SHAP analysis in predictive modeling (Fig. 9A–F).

## 4. Discussion

The growing potential of machine learning, particularly gradient boosting algorithms like supervised CatBoost, in forecasting the risk of thyroid cancer recurrence is highlighted by this study’s findings. The model achieved an impressive 97% accuracy and an AUC of 0.99 on the test dataset, underscoring its robust predictive capacity. This result aligns consistently with prior machine learning applications in oncology, where tree-based ensemble methods frequently deliver high accuracy and AUC values. Furthermore, the competitiveness of advanced tree-based methods in managing complex clinical datasets is demonstrated by the close performance alignment of supervised CatBoost with other top-performing models, such as Extra Trees, Light GBM, and XGBoost. Although these high-test set metrics suggest strong generalization, the possibility of overfitting was considered; however, as the reported success stems from the test dataset, the risk appears minimal, reinforcing the model’s reliability for clinical predictions, pending further validation on additional unseen data.

The integration of SHAP values for interpretability is presented as one of the distinctive advantages of this work. Although high accuracy is essential, a transparent understanding of the model’s rationale is also required for clinical decision-making. It was revealed by the global SHAP analysis that factors such as Response, Risk, and N (lymph node involvement) exert the greatest influence on recurrence predictions. Alignment with established clinical knowledge is indicated by these findings, which show that high-risk categories, lymph node metastasis, and suboptimal responses to therapy are strong indicators of disease relapse. Moreover, the nuanced ways in which varying degrees of treatment efficacy and risk stratification affect recurrence likelihood are specifically highlighted by the dependence plots for *Response* and *Risk*, thus offering clinicians actionable insights.

Further granularity is provided by local SHAP analyses by illustrating how specific features drive individual predictions, whether correct or incorrect. The model’s capacity to synthesize multiple clinical and demographic variables such as tumor stage, age, and multifocality into a cohesive recurrence assessment is validated by the examination of TP and TN cases. Meanwhile, cautionary tales are provided by FP and FN instances. In some cases, other risk indicators were overshadowed by an overreliance on a single feature (e.g., an especially high weighting on Response), resulting in misclassification. The recognition of these pitfalls is crucial for model refinement and underscores the importance of a balanced, multi-feature approach.

From a clinical standpoint, paramount importance is ascribed to the interpretability of these results. The exact features that lead to a model’s prediction can be pinpointed, which allows the algorithm’s decision-making to be validated by healthcare providers in the context of individual patient profiles. For example, if a patient is flagged as high-risk predominantly due to Age and Risk category, other relevant clinical indicators such as imaging results and laboratory values can be double-checked by clinicians to either confirm or question the algorithm’s suggestion. More personalized care pathways, better treatment planning, and potentially improved patient outcomes can be facilitated by this synergy between machine learning outputs and expert human interpretation.

An important advantage of our approach lies in its ability to combine predictive performance with clinical interpretability addressing a common shortcoming of traditional black box models. Through SHAP analysis, each variable’s individual contribution to the model’s decision can be assessed, enabling clinicians to understand and trust the rationale behind recurrence predictions. This transparency is particularly valuable in oncology, where treatment decisions must be personalized and evidence based. However, 1 limitation stems from the relatively small sample size of 383 patients, which may restrict the generalizability of findings to broader, more diverse populations. In datasets with class imbalance or limited representation of certain subgroups, SHAP derived feature importance values may reflect underlying distributional bias, potentially leading to misleading interpretations. Therefore, while the interpretability of our model is a clear strength, it also requires cautious application and further validation using larger, multi-institutional datasets to ensure robustness and reduce the risk of biased conclusions.

Although categorical features are adeptly handled by a supervised CatBoost, and high accuracy was demonstrated, the potential benefits of an ensemble or hybrid approach could be overlooked by reliance on any single algorithm. Additionally, external validation on larger, more diverse cohorts is necessary to assess generalizability, even though the dataset is relatively comprehensive. The distribution of features such as age, gender, or specific tumor histological variants may differ in other populations, and model performance may potentially be affected. The evidence bases for adopting such models in routine clinical practice would be strengthened by further research incorporating multi-institutional or global datasets, as well as prospective trials.

Finally, the future trajectory of explainable AI in healthcare is situated in its capacity to combine predictive accuracy with clinically meaningful transparency. Both the precision and interpretability of these algorithms could be enhanced by advances in model-agnostic explanation tools and more refined data collection protocols. As molecular markers, genetic profiles, and real-time monitoring data become increasingly integrated into patient records, the explanatory power of models like CatBoost can be expected to grow, thereby further revolutionizing early detection and recurrence risk assessment in thyroid cancer.

## 5. Conclusion and future work

The present study has illustrated the effectiveness of leveraging a supervised CatBoost classifier for predicting thyroid cancer recurrence, underscoring the value of integrating clinical, demographic, and pathological variables. High predictive accuracy and robust performance metrics were achieved, thereby reinforcing the potential of machine learning in refining risk assessments for WDTC. The incorporation of SHAP values yielded significant interpretive benefits, enabling a clearer understanding of the rationale behind model predictions. By pinpointing dominant features such as Response, Risk, and *N*, it has been shown that the alignment between the model’s high-impact variables and established clinical knowledge is strong, further enhancing confidence in the model’s applicability. Moreover, local SHAP analyses have offered granular insights into individualized cases, thereby facilitating refined decision-making and targeted clinical interventions.

Despite these positive outcomes, certain limitations warrant further expansion. The reliance on a single algorithm, although effective, may not fully capture the advantages of ensemble or hybrid approaches, especially for complex disease presentations. Moreover, additional validation on large-scale and diverse datasets is recommended to confirm the model’s generalizability beyond the current cohort. Future research should seek to integrate additional molecular and genetic data, aligning with the broader trend toward precision medicine. Ongoing improvements in model-agnostic explanation tools and more granular data collection protocols also hold promise for enhancing the accuracy and interpretability of predictive models. By addressing these directions, a more comprehensive and actionable framework for thyroid cancer recurrence risk stratification can be developed, ultimately contributing to improved patient outcomes.

## Acknowledgments

The authors would like to thank researchers who publicly share their dataset in the UCI Machine Learning Repository. Also, the authors acknowledge the An-Najah National University in Palestine (www.najah.edu) and Sakarya University of Applied Sciences for the technical support provided to publish the present manuscript.

## Author contributions

**Conceptualization:** Ahmad A. Hanani, Turker Berk Donmez, Mustafa Kutlu, Mohammed Mansour.

**Data curation:** Turker Berk Donmez, Mustafa Kutlu, Mohammed Mansour.

**Formal analysis:** Turker Berk Donmez, Mustafa Kutlu, Mohammed Mansour.

**Methodology:** Ahmad A. Hanani, Turker Berk Donmez, Mustafa Kutlu, Mohammed Mansour.

**Project administration:** Ahmad A. Hanani, Mustafa Kutlu, Mohammed Mansour.

**Software:** Mustafa Kutlu, Mohammed Mansour.

**Supervision:** Ahmad A. Hanani.

**Writing – original draft:** Ahmad A. Hanani, Turker Berk Donmez, Mustafa Kutlu, Mohammed Mansour.

**Writing – review & editing:** Ahmad A. Hanani, Turker Berk Donmez, Mustafa Kutlu, Mohammed Mansour.
